# Stroke survivors' and informal caregivers' experiences of primary care and community healthcare services – A systematic review and meta-ethnography

**DOI:** 10.1371/journal.pone.0192533

**Published:** 2018-02-21

**Authors:** Dominika M. Pindus, Ricky Mullis, Lisa Lim, Ian Wellwood, A. Viona Rundell, Noor Azah Abd Aziz, Jonathan Mant

**Affiliations:** 1 Primary Care Unit, Department of Public Health and Primary Care, University of Cambridge, Strangeways Research Laboratory, Worts’ Causeway, Cambridge CB1 8RN, United Kingdom; 2 Department of Family Medicine, National University of Malaysia, Bandar Tun Razak Cheras, Kuala Lumpur, Malaysia; Northwestern University Feinberg School of Medicine, UNITED STATES

## Abstract

**Objective:**

To describe and explain stroke survivors and informal caregivers’ experiences of primary care and community healthcare services. To offer potential solutions for how negative experiences could be addressed by healthcare services.

**Design:**

Systematic review and meta-ethnography.

**Data sources:**

Medline, CINAHL, Embase and PsycINFO databases (literature searched until May 2015, published studies ranged from 1996 to 2015).

**Eligibility criteria:**

Primary qualitative studies focused on adult community-dwelling stroke survivors’ and/or informal caregivers’ experiences of primary care and/or community healthcare services.

**Data synthesis:**

A set of common second order constructs (original authors’ interpretations of participants’ experiences) were identified across the studies and used to develop a novel integrative account of the data (third order constructs). Study quality was assessed using the Critical Appraisal Skills Programme checklist. Relevance was assessed using Dixon-Woods’ criteria.

**Results:**

51 studies (including 168 stroke survivors and 328 caregivers) were synthesised. We developed three inter-dependent third order constructs: (1) marginalisation of stroke survivors and caregivers by healthcare services, (2) passivity versus proactivity in the relationship between health services and the patient/caregiver dyad, and (3) fluidity of stroke related needs for both patient and caregiver. Issues of continuity of care, limitations in access to services and inadequate information provision drove perceptions of marginalisation and passivity of services for both patients and caregivers. Fluidity was apparent through changing information needs and psychological adaptation to living with long-term consequences of stroke.

**Limitations:**

Potential limitations of qualitative research such as limited generalisability and inability to provide firm answers are offset by the consistency of the findings across a range of countries and healthcare systems.

**Conclusions:**

Stroke survivors and caregivers feel abandoned because they have become marginalised by services and they do not have the knowledge or skills to re-engage. This can be addressed by: (1) increasing stroke specific health literacy by targeted and timely information provision, and (2) improving continuity of care between specialist and generalist services.

**Systematic review registration number:**

PROSPERO 2015:CRD42015026602

## Introduction

Globally, stroke is the second leading cause of death and the third most important cause of disability burden.[1, 2] Stroke-related disability burden is on the rise with a 12% increase worldwide since 1990. This rise accounts for more than 100 million Disability Adjusted Life Years (DALYs) lost (> 2 million in the USA alone, 0.66 in the UK) and contributes to the large economic burden of stroke due to healthcare utilisation, informal care and the loss of productivity (for example, DALYs of younger stroke survivors (<75 years old) account for 70% of DALYs lost).[1, 3] The cost of stroke is high and estimated at $33 billion (including health care cost, medicines and missed days of work)[4] in the USA, £8.9 billion per annum in the UK[3] and $5 billion in Australia (including healthcare, informal care and the loss of productivity).[5]

Primary care could play an important role in the care of stroke survivors and their caregivers, supporting access to community services, facilitating transfer back to specialist services when new problems emerge, providing training, respite care, and identifying and addressing health needs of caregivers, and managing those aspects of care that are traditionally managed in general practice (for example, risk factors and psychological issues). However, the feeling of abandonment that people with stroke experience following hospital discharge suggests this role is not being completely fulfilled.[6–8] Qualitative reports indicate that lack of co-ordinated post-discharge care leaves patients and informal caregivers feeling unsupported.[6, 8–12]

No comprehensive systematic review of stroke survivors’ and caregivers’ specific experiences of primary care and community healthcare services has been performed. Previous qualitative reviews offer a broader focus including experiences of acute services[13] or are more selective, omitting research focussed on specific problem areas (e.g. information provision[14]). Meta-ethnography may offer new insight into how post-discharge care after stroke could be improved by providing a conceptual framework which surpasses simple aggregation of primary findings.[15] Our aim was to synthesise qualitative evidence on community-dwelling stroke survivors’ and caregivers’ experiences of primary care and community healthcare services after stroke in order to explain where these experiences originate from and how they could be addressed by healthcare services and interventions.

## Methods

### Search strategy

A review protocol has been previously published[16] (PROSPERO 2015:CRD42015026602). Searches of four electronic databases (Medline, CINAHL, Embase and PsycINFO) were conducted by NAZ and VH between May and June 2015 (see appendix A) using PICo mnemonics[17]:

- **P**opulation included stroke survivors and family caregivers
- **I**nterest focused on experiences, perspective, satisfaction and needs
- **Co**ntext included primary care, community health services and general practice

Caregivers were defined as unpaid carers, including spouse or partner, family members, friends, or significant others who provide physical, practical, transportation or emotional help [16]. Keywords relevant to the study type (qualitative, interview, focus group) were included. No date, language or country restrictions were applied, but we did not include non-English language papers in the synthesis; references of eligible articles were checked for relevance.

### Study selection and data collection

We included studies that used qualitative methods of data collection and analysis, and described the experiences of adult (≥ 18 years old) community-dwelling stroke survivors and/or informal caregivers of primary care and community healthcare services after stroke.[16] Community healthcare services included district nurses, community rehabilitation services (e.g., physiotherapy (PT), occupational therapy (OT))[18], speech and language therapist (SLT) and clinical psychology. We excluded papers which: 1) used mixed-methods designs where the qualitative data could not be separated; 2) included multiple patient populations; 3) focused on the in-patient setting, nursing or residential homes, or 4) multiple settings (for example, hospital, early supported discharge, nursing homes, community setting) and did not distinguish between them in their analyses.[16] Descriptive data, quality assessment, themes identified by the authors and their description using author’s original language or a paraphrase were recorded for each paper in a Microsoft Excel spreadsheet by two independent reviewers.

### Quality appraisal

Quality of included studies was assessed using the Critical Appraisal Skills Programme Qualitative Research Checklist (CASP)[19] by two reviewers. Studies could achieve a maximum score of 10 points. We did not exclude studies based on CASP quality assessment, but used this as descriptive information to add to the critical analysis. Studies were rated for relevance using criteria introduced by Dixon-Woods et al.[20] This divided papers into four categories in relation to our research objectives. Firstly, key papers, were conceptually rich with potential to make an important contribution to the synthesis; Secondly, satisfactory papers had potential value to the synthesis. Studies which were deemed irrelevant to our objectives or to have fatal methodological flaws were excluded. A fatal flaw was a subjective assessment by the reviewer that the methodology was so poor that it was not appropriate to make use of the results. A third reviewer was consulted when consensus could not be reached.

### Synthesis

We used meta-ethnography to synthesise qualitative studies.[21] Meta-ethnography is particularly well-suited to provide new insights into understanding the experiences of stroke survivors and their caregivers as it provides a conceptual framework which surpasses simple aggregation of primary findings.[15, 22] We adopted a paradigm neutral approach and synthesised studies based on their thematic rather than theoretical similarity.[23, 24]

**Fig 1:**
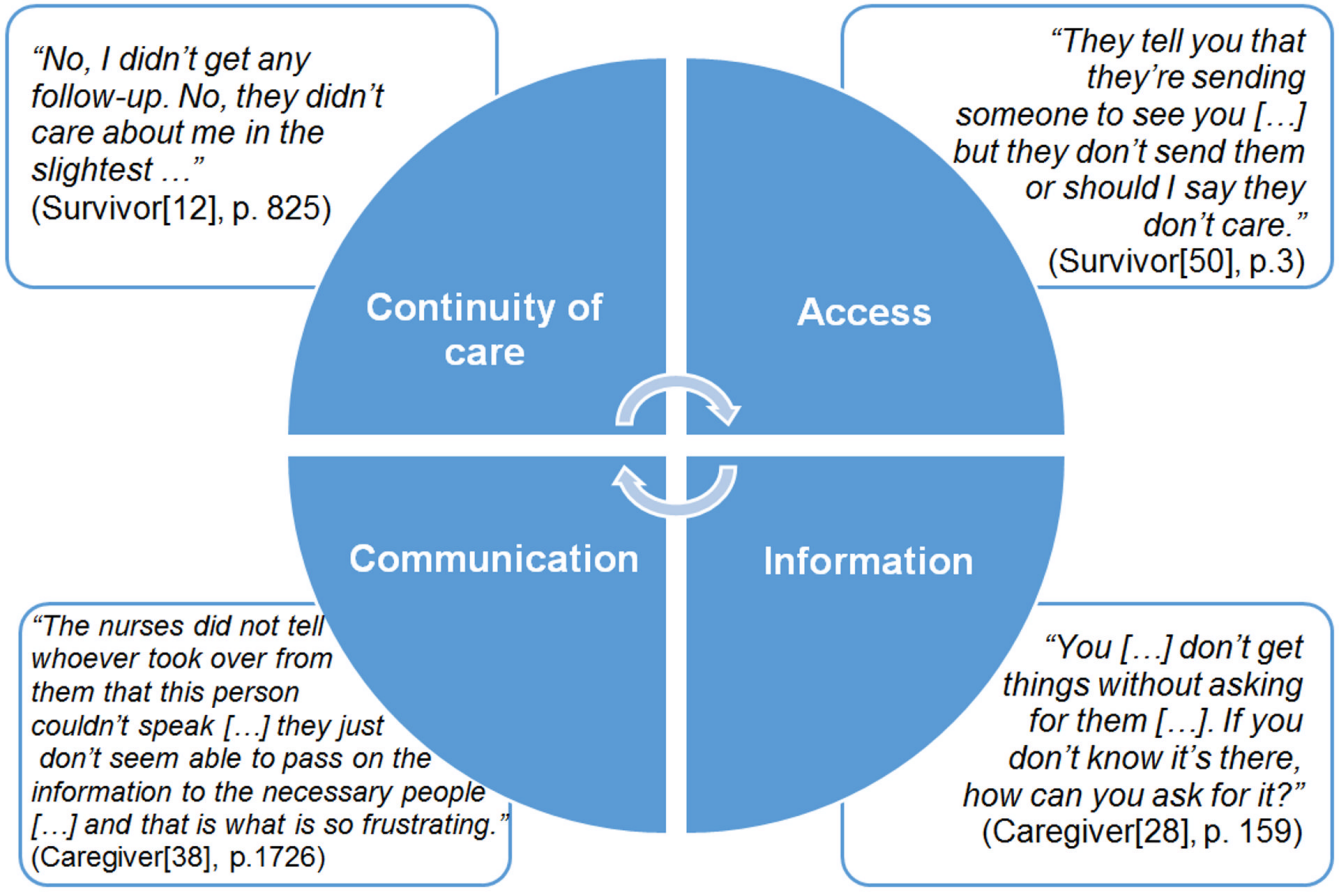
Inter-relationships among categories of second order constructs.

Our unit of analyses were themes identified by the authors of included studies (i.e. second order constructs reflecting authors’ interpretations of primary data, namely participants’ accounts which constituted first order constructs[15]). Three groups of two reviewers (DMP, NA, VH, AVR, IW, RM, LL) read a subset of papers and recorded second order constructs with brief descriptions in the authors’ own words. Contextual details were recorded (aim, country of origin, sample characteristics, study setting, data collection and analysis methods). DMP and RM interpretatively read and compared second order constructs across the studies, and summarised them into second order constructs shared by the studies (Table 1). The summary included descriptions “that had a meaning for all the studies [which included a relevant theme]” ([15], p. 161). We then grouped the second order constructs into categories reflecting key characteristics of post-discharge care: (1) continuity of care, (2) access to services, (3) information and (4) quality of communication (Fig 1).

Finally, we developed third order constructs which represent our own explanation of why stroke survivors’ and caregivers’ had the experiences of primary care and community healthcare services that were represented in the second order constructs. We used the second order constructs as building blocks to develop these third order constructs and to make a ‘line of argument synthesis’ in which we could consider how the findings might inform a sustainable model of long term support [16] The process was iterative and involved a consultation exercise with qualitative and healthcare researchers with expertise in primary care and stroke.

**Table 1:** Second and third order constructs.

Third order constructs		Second order constructs		Papers
**Perceived marginalisation**				
		**Continuity of care**		
		The need for greater continuity of care	Lack of support and feeling abandoned; no individual plan; waiting time between hospital discharge and initiating therapy in the home. Caregivers stress the need for ongoing support (essential) and felt that the stroke survivor was going backwards while waiting for community rehab services. Caregivers also wanted to know how they could initiate some form of rehabilitation. Absence of longer term reassessment by allied healthcare professionals was apparent, however some survivors were generally satisfied and very appreciative of services of PTs, OTs, and speech and language therapists (SLTs). Those with improvements had no opportunity to access further therapeutic advice. Significant gaps in services provided while longer rehabilitation (PT) was wanted. Little help from social or specialist services or little contact with the hospital.	[8–10, 27, 28, 36, 38, 39, 47, 54, 55]
		**Access**		
		Lack of much needed support	Limited or lack of support in all areas e.g. help to get organised and establish a routine; emotional and social support; insufficient or quickly diminished support. Levels of support should be maintained throughout the caregivers’ career; worries about what might happen if caregiver gets ill; felt they had to become experts in caring role.	[**6, 25**–28]
		Caregivers’ perceptions of a good therapy service	A good therapy has to bring an improvement in physical functioning of stroke survivors. The lack of individualised treatment perceived as a problem by both a stroke survivor and a caregiver. Dissatisfaction with quality of services, for example, too low intensity of community inpatient rehabilitation after discharge, rehabilitation was unspecific (mostly included walks); dissatisfaction with healthcare professional-patient and family communication which was perceived as too negative.	[12, 29–32]
		Understanding of individual needs	Services did not always understand the person with stroke as an individual, some preferred to access culturally specific or mainstream services, not always acknowledged by services	[33]
		Exercise potentially beneficial but access to physiotherapy preferred	Exercise (e.g. exercise referral scheme, a part of a secondary prevention programme with goal setting) brought physical and psychological improvements, increased physical activity (PA), fitness, strength and movement (ER), and improved ability to do activities of daily living (ADLs) plus increased participation (Masterstroke programme). However, survivors want more individualised care, i.e. more PT rather than exercise in the gym.	[34–36]
		Lack of vocational support to return to work	Younger survivors (mean age 49 years) were very disappointed with the lack of support from community services to return to work.	[37]
		Caregivers’ need for training*	Little preparation given to caregivers in relation to the hospital discharge of a stroke survivor which created panic and anxiety. Training mostly needed in practical caring skills, information on available support, help with form filling, training in advocacy skills and looking after their own health.	[**6**, 9, 25, 38–40]
		Back-up and respite services for caregivers	Caregivers felt they needed support from services, including back-up services in emergencies and respite services. Services which provided respite included day hospital care; lack of resources in the community and the difficulty in identifying and accessing available resources as important barriers to continuing in the caregiving role. Caregivers’ state of health when planning support service needs to be considered.	[8, 9, **25**, 28, 29, 40]
		Help for caregivers from voluntary agencies and peers	Voluntary agencies important sources for information and equipment in the immediate post discharge phase. Support groups and a 12 week peer delivered intervention can contribute to decreased burden.	[**6**, **41**, **42**]
		**Information**		
		Information on stroke, its consequences and recovery	Information on stroke and risk of future stroke is of key importance. More education and explanations on stroke from healthcare professionals requested. Information on causes of stroke, effects of stroke and treatment decisions needed. When information provided (e.g. as part of living with Dysarthria or Masterstroke programmes) it was listed as a benefit. Sometimes information difficult to deal with as it brought back memories of stroke. More (and on-going) information needed on the trajectory of recovery and prognosis. Survivors questioned if the onset of strokes could have been prevented if healthcare professionals had had appropriate knowledge about stroke.	[25, 35, 37–39, **41**, 43, **44**–48]
		Information on secondary prevention and concerns about medication	Information on secondary prevention was a priority. More information on secondary prevention and how to prevent another stroke wanted but little or no information received (e.g. in hospital). Some survivors did receive general lifestyle information including diet and exercise, most in written format and not reinforced verbally. Survivors had concerns about taking medication and its adverse effects and interactions. Concerns were reduced when no adverse effects experienced.	[11, 27, 49–51]
		**Communication**		
		Ineffective communication between healthcare providers, patient and family*	Systems of communication with a patient after discharge are necessary. Gaps in the transfer of knowledge between healthcare professionals were highlighted. Insufficient explanations about the treatment. Caregivers felt lack of confidence to speak to healthcare professionals. Language used to describe diagnosis caused confusion. Clinicians need to communicate effectively.	[29, 33, 38, **44**, 47, 49, 52]
		Quality of the relationship with healthcare professionals important†	Sympathy, empathy and understanding were valued. Healthcare professionals who seemed to be doing all they could and were easily approachable were valued. Having confidence in personnel and being a part of the planning of continued care important.	[12, 28, 53]
**Passivity/activity**				
	**Continuity of care**			
	Dissatisfaction with the lack of follow-up and need for formal support*		Caregivers were disconcerted by the lack of hospital or a GP follow-up; they felt that stroke survivor was forgotten or written off. Dissatisfaction with the lack of monitoring from the healthcare system. Caregivers used stroke services, appreciated regular check-ups as reassurance.	[**6**–8, 10–12, **25**, 29, 47]
	Healthcare professionals who could facilitate continuity of care		GP, Family Support Organizer, social services, rehabilitation services (e.g. PT).	[**6**, 10, 11, 54, 55]
		**Access**		
		Support from healthcare professionals and community services facilitates recovery and social participation*	Survivors look for guidance in physical recovery, support with psychological, emotional or social issues, but little professional support was available. Healthcare professionals’ support at home as part of the improvements goal programme (including self-management and self-monitoring) was perceived as essential in improving self-care. Domiciliary rehabilitation services provided convenience and comfort, caregiver education and rehabilitation process geared towards their home environment. Community services (health & social services, community organisations) can act as either important facilitators or as barriers to social participation of survivors with aphasia.	[37, **44**, 45, 56, 57]
		Limited access*	Access to healthcare was jeopardized because of geographic distance or transportation difficulties. Another limiting factor was a mismatch between survivors’ expectations (e.g. community rehabilitation to address support with rehousing, transport, management of stress, emotional and interpersonal difficulties) and the remit of service. Therapy was needed earlier and for longer (e.g. PT, OT, SLT). Timing of home care services was crucial and the main reason to stop accessing the service even when it was felt that the service was needed.	[37, 38, 58]
		Difficulties accessing health services	Delayed access or problems accessing services was highlighted; these included: rehabilitation services (PT, OT, SLT), social services, home care, equipment, supplies and financial assistance. Difficulty accessing appointments, cancellations, visits were too short. Navigating the field of community stroke care difficult. However, when early and continuing rehabilitation services were available, survivors appreciated practical advice from therapists during early rehabilitation.	[8, 26, 27, 37–39, 43, 50, 52, 59]
		Community services much needed	The need for community services: home visit from healthcare personnel, domestic help, municipal nurse, (attendance at day centre). Not enough financial support and social services and psychological services. Services received from PTs, OTs and SLTs generally very appreciated. Services of Family Support Organizer were appreciated and provided regular check-ups as reassurance; continuity in knowledge and a valuable resource to turn to if things became “too much”.	[39, 40, 43, 55, 60, **61**]
		Help needed with benefits*	Problems completing benefit forms within deadlines for benefits agencies; knowing about how to apply for help with a problem. Information and help from a variety of sources (social services and voluntary agencies) was perceived as helpful.	[**6**, 28, 55]
	**Information**			
	Information on availability and access to services*		More and increasingly detailed information needed on what services are available in the community and how to access them. Services listed included: benefits, home adaptations, support groups, home help, equipment and the information on the roles of healthcare providers (e.g. differences between an OT and a social worker). Both survivors and caregivers were unaware of local stroke support groups which were potentially very important in helping them adjust. Caregivers wanted information on stroke associations, caregiver support groups, home help, future rehabilitation options or services, and community support facilities (e.g. hydrotherapy).	[10, 27, 28, 48, 55, 59, 62]
	Methods of accessing information*		Uncertainty on how to access information. Caregivers accessed information from various sources: books, a doctor, leaflets from caregiver group, gained by chance (e.g. in a conversation). Survivors mentioned internet or voluntary organisations as sources.	[25, 37, 55]
	Information format		Format of the information needs to be considered: both written and verbal information needed; written format not appropriate for survivors with aphasia. Courses by stroke unit and stroke groups perceived as highly relevant. Other useful formats of information provision: telephone contact with healthcare professional, drop in centre, face-to-face contact (preferred to telephone line for emotional support). Importance of availability, quick responses and personalised information.	[9, 11, 28, 38, 39, 51, 62]
	The need for a coordinated information resource*		A single route to information, services, and practical help, whatever the problem, would make life easier. Having a resource folder as part of the course (Living with Dysarthria) was perceived as a fantastic resource. Caregivers felt that information was available but that they would need to know where to seek it and what to request. Caregivers accessed information from various sources: books, a doctor, leaflets from caregiver group, gained by chance (e.g. in a conversation). Survivors mentioned internet or voluntary organisations. Navigation of the healthcare system was difficult, as was knowing what resources were available and how to access them.	[25, 28, 45, 62]
	Education as potential motivator to adherence to a lifestyle change		Patients struggle to adhere to treatment regimens (e.g. not drinking alcohol) despite encouragement from healthcare professionals. Reasons included: 1) questioning the value of healthy lifestyle (as it did not prevent stroke or due to changing information from health campaigns), or 2) home help service not facilitating healthy eating. For caregivers the perceived stress-relieving properties of alcohol and tobacco were a barrier to healthy lifestyle. However, education about diet and nutrition within a self-management programme could act as a motivator.	[35, **44**, 51]
	Preferred information provider		More information from healthcare professionals in general and a GP in particular. When information was provided by nurses, social workers and PTs, most caregivers (#12) were satisfied with information. PT; some preferred that information was provided by a doctor.	[10, 28, **44**]
**Change and fluidity of needs**				
	**Continuity of care**			
	Proactive follow-up expected from a GP†		Caregivers expected immediate and automatic GP follow-up for at least one year after discharge. In practice, very few caregivers reported GP follow-up. Some reported a total lack of contact with their GP. Routine contact with primary care would be appreciated. Many satisfied with the support from GP and practice nurses but some were disappointed with the lack of support from GP. Some perceived services as reactive. Caregivers usually described the contact with GP practice in relation to a stroke survivor rather than to themselves.	[**6**, 9, 11, 27, 28, 39]
	Need for ongoing support from healthcare professionals†		Caregivers voiced the need for ongoing support also during adaptation phase (several months after stroke). However, none received any long term information and healthcare professionals did not discuss caregivers’ long term support needs.	[**63**]
	Support needed with medication adherence at the time of transition to home†		Caregivers’ role in medication management; non-adherence at the point of transition from hospital to home due to forgetting, complex regimen, night-time dose.	[11]
	**Information**			
	Timing of the information		Information giving must be appropriate to the stage of recovery. Caregivers felt information was provided not in the right time. For example excess information in first few weeks, comments that they did not know what questions to ask at that point, and there had been no follow-up opportunity. Stroke survivors reported the need for information after the acute phase due to the difficulty in processing information at that time.	[6, 11, 28, 49, 51, 59, 63]

* Themes relating to both perceived marginalisation and passive and active services

† Themes relating to change and the fluidity of needs as well as passive and active services. Key papers are marked in bold.

## Results

**Fig 2:**
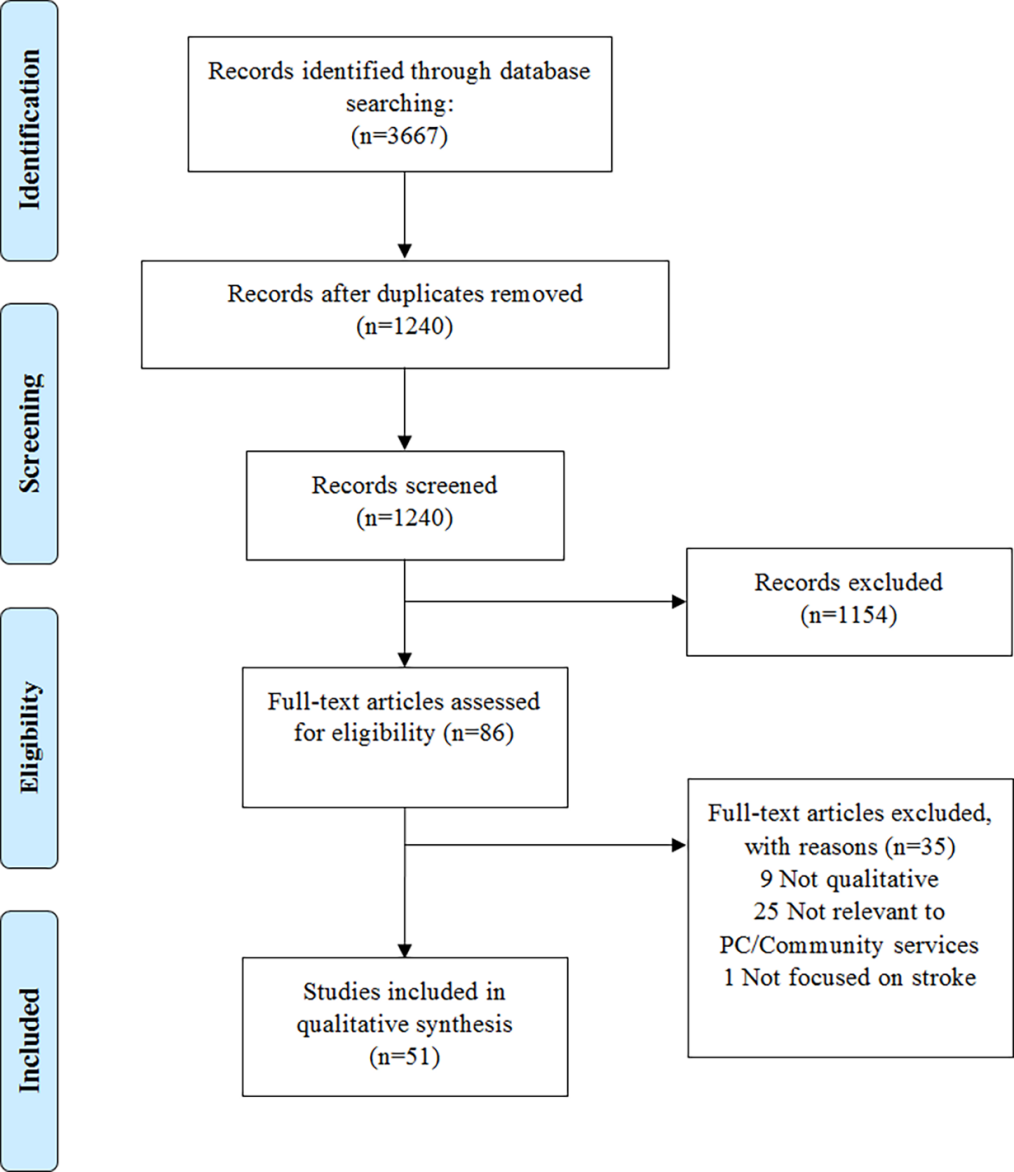
PRISMA flow diagram of studies included and excluded at each stage of the review.

We identified 3,667 potentially relevant articles. After excluding duplicates, title and abstract screening, 86 full reports were read in full and assessed for eligibility. 51 papers representing 51 unique studies including 496 participants (168 stroke survivors and 328 informal caregivers) were included in the final synthesis (Fig 2).

Study and participant characteristics are listed in Table 2. Almost half of studies (n = 20) included both stroke survivors and informal caregivers, and 17 studies included survivors from across the stroke continuum (within the first year after stroke n = 12, and beyond n = 12). Studies originated from the UK (25), North America (12), Australia (8) and Scandinavia (5), and one study was from Iran.[43] The majority of studies (n = 32) used interviews; eight used focus groups.

**Table 2:** Characteristics of the reviewed studies.

Citation	Ref.	Country	Survivor / Caregiver	Participant characteristics Survivor / Caregiver n (n females), age range	Analytical method
Allison 2008	[49]	England	Both	25 (11) 37–91 years/13 (8) NR	Framework
Barnsley 2012	[64]	Australia	Survivors	19 (7) years	Open/axial coding
Blixen 2014	[52]	USA	Both	10 (0) 34–64 years/7 (7) 49–61 years	Constant comparative method
Brown 2011	[65]	Australia	Caregivers	24 (15) 40–87 years	Interpretive phenomenological analysis
Brunborg 2014	[60]	Norway	Survivors	9 (5) 61–96 years	Thematic coding
Burman 2001	[66]	USA	Both	13 (8) 28–85 years	Constant comparative method
Cameron 2013	[63]	Canada	Caregivers	24 (17) 36–77 years	Framework
Cecil 2011	[38]	Northern Ireland	Caregivers	10 (10) NR	Inductive method
Cecil 2013	[39]	Northern Ireland	Caregivers	30 (23) 36–84 years	Inductive method
Dalvandi 2010	[43]	Iran	Survivors	10 (4) 55–70 years	Open/axial/selective coding
Danzl 2013	[61]	USA	Both	13 (9) 42–89 years/12 (7) 38–75 years	Content analysis
Denman 1998	[25]	England	Caregivers	9 (6) NR	Thematic analysis
Donnellan 2013	[44]	Northern Ireland	Survivors	8 (2) 52–83 years	Content analysis
Dorze 2014	[56]	Canada	Survivors	19 (5) 51–84 years	Narrative analysis
Eaves 2002	[58]	USA	Both	8 (6) 56–79 years/18 (6) 21–70 years	Interpretive phenomenological analysis
El Masry 2013	[29]	Australia	Both	10 (2) 41–90 years/20 (16) 31–90 years	Thematic analysis (Kruger’s method*)
Gosman-Hedstrom 2012	[67]	Sweden	Caregivers	16 (16) 74–86 years	Constant comparative method
Grant 1996	[26]	USA	Both	10 (NR) 45–82 years/10 (9) 32–68 years	Inductive method
Graven 2013	[68]	Australia	Both	8 (2) 58–89 years/6 (5) 49–75 years	Thematic analysis
Greenwood 2011	[9]	England	Caregivers	13 (8) NR	Constant comparative method
Hare 2006	[27]	England	Both	27 (13) 43–88 years/6 (6) NR	Thematic analysis
Hart 1999	[54]	England	Both	57 (25) ~65–85 years	Framework
Jones 2008	[50]	England	Both	35 (NR) 25–92 years/20 (NR) NR	Thematic analysis
Law 2010	[59]	Scotland	Survivors	14 (6) 33–76 years	Framework
Lawrence 2010	[51]	Scotland	Both	29 (13) 37–81 years/20 (9) 42–79 years	Thematic analysis
Lilley 2003	[55]	England	Survivors	20 (NR) Mean = 63 years	Content analysis
Low 2004	[30]	England	Caregivers	40 (29) Mean = 68 years	Thematic analysis
Mackenzie 2013	[45]	Scotland	Both	12 (5) 50–93 years/7 (7) NR	Hermeneutic phenomenological
Martinsen 2015	[8]	Norway	Survivors	14 (5) 21–67 years	Framework
Reed 2010	[31]	England	Survivors	12 (7) NR	Constant comparative methods
Saban 2012	[41]	USA	Caregivers	46 (46) 18–73 years	Giorgi’s method (phenomenology)
Sabari 2000	[32]	USA	Both	6 (1) 45–75 years/4 (4) 45–75 years	Constant comparative method
Sadler 2014	[37]	UK	Survivors	31 (12) 24–62 years	Content analysis
Sharma 2012	[34]	England	Survivors	9 (4) 37–61 years	Constructivist qualitative approach
Simon 2002	[28]	England	Both	8 (NR) NR/NR	Framework
Ski 2007	[10]	Australia	Both	13 (8) 59–84 years/13 (6) 42–81 years	Content analysis
Smith 2004	[6]	Scotland	Caregivers	90 (65) 19–84 years	Thematic analysis
Souter 2014	[11]	Scotland	Both	30 (15) 32–86 years/8 (NR) years	Framework
Stewart 1998	[42]	Canada	Caregivers	20 (20) NR	Inductive method
Strudwick 2010	[33]	England	Caregivers	9 (8) 30–72 years	Inductive method
Talbot 2004	[40]	Canada	Both	4 (NR) 71–85 years/5 (NR) 41–69 years	Categorised according to the Handicap Production Process
Taule 2015	[53]	Norway	Survivors	8 (4) 45–80 years	Interpretative Description*
Tholin 2014	[12]	Sweden	Survivors	11 (5) 49–90 years	Content analysis
Tunney 2014	[7]	Northern Ireland	Caregivers	10 (10) NR	Thematic analysis
van der Gaag 2005	[57]	England	Both	38 (12) 31–81 years/22 (16) 36–81 years	Matrix based method
White 2007	[62]	Canada	Caregivers	14 (NR) NR	Inductive method
White 2009	[47]	Australia	Survivors	12 (6) 43–92 years	Inductive method
White 2013	[35]	Australia	Survivors	9 (2) 53–80 years	Content analysis
White 2014	[46]	Australia	Survivors	8 (2) 69–88 years	Constant comparative method
Wiles 1998	[48]	England	Both	9 (10) 50–85 years / 12 (NR) NR	Thematic analysis
Wiles 2008	[36]	England	Survivors	9 (1) 18–78 years	Thematic analysis

*Note*. *F*: females; *NR*: Not reported

*Kreuger’s method: descriptive and interpretative analysis of focus groups; *Interpretative Description*: analysis focused on meaning to generate knowledge of individuals’ experience.

### Critical appraisal

All but two studies[26, 66] scored 7 or above on the CASP Qualitative Checklist. None were assessed as being fatally flawed. Main quality limitations pertained to lack of consideration of the relationship between participant and researcher (19 studies), rigour of the data analyses (6 studies), and consideration of ethical issues (4 studies). We identified 7 key papers.[6, 25, 41, 42, 44, 61, 63]

### Theoretical standpoints

Few studies specified a theoretical approach. Those which did, either adopted Grounded Theory[43, 48, 55, 64, 66] or phenomenology.[8, 29, 31, 32, 34, 60, 65] A variety of analytical methods were reported (Table 2).

### Synthesis of findings

#### Second order constructs

The first second order construct (continuity of care) included follow-up after hospital discharge[6, 7, 9–12, 27, 47] and facilitation by healthcare professionals.[6, 11, 42, 54, 55] An unaddressed need for continued support was voiced in a quarter of studies.[6–12, 27, 28, 47, 54, 55, 61] Survivors and caregivers felt frustrated and dissatisfied with a lack of proactive follow-up either from primary care,[6, 9, 11, 12, 27] the hospital,[7, 10] or allied healthcare professionals.[47] This led to feelings of dissatisfaction,[8] uncertainty,[8, 10, 47] that a stroke survivor was “forgotten and written off”[25] and that their general practice did not care about them.[12] When regular follow-up was provided, survivors felt supported.[12]

The next second order construct related to access to services. Stroke survivors expected support from community rehabilitation with rehousing, transport, management of psychological and interpersonal difficulties, but these were outside the remit of the services.[37] Although generally appreciated, rehabilitation (PT, OT) was often perceived as insufficient and prematurely withdrawn.[38, 39] Survivors and caregivers felt more progress could have been achieved with longer therapy.[38, 39]

When support from community healthcare services (e.g. SLTs, nurses) was offered either through specifically designed programmes or community organisations targeting survivors with specific needs (dysarthria or aphasia), these were generally appreciated and resulted in feelings of confidence,[45, 57] reassurance,[56] and encouraged positive coping behaviours.[45, 56] Participation in community organisations for people with aphasia gave a sense of belonging, protection and reduced worries.[56]

Emotional support was deemed important but lacking for both survivors and caregivers. Although anxiety and the lack of confidence were common, often survivors did not seek professional help.[27] Having someone with whom to discuss difficulties and who provided motivation was considered valuable to reduce feelings of depression.[8] A sub-group of younger stroke survivors felt disappointed with the lack of vocational support to return to work, contributing to their financial hardship, disappointment,[37] and feelings of loss.[35]

Lack of support for caregivers was reported in 11 studies.[6, 8, 9, 25–29, 38–40] Caregivers felt healthcare professionals assumed that they would provide the majority of care needed[27], with little or no support.[6, 9, 25–27, 29, 38–40] They felt ill prepared and pressured to “become experts” in caring for stroke survivors[27]; causing them anxiety.[39] The need for training was repeatedly emphasised.[6, 9, 25, 38] Caregivers wanted insights into how to cope,[38] how to get organised and establish a routine after discharge.[25] Many also wanted back-up[25, 28, 40] and respite services.[8, 9, 40, 67] Lack of support was highlighted as a barrier to undertaking and/or continuing the caregiving role.[62]

Long waiting times for assessment and rehabilitation,[10, 54] and little or no help from social services[8, 54] left survivors feeling “left in the lurch”.[8] Caregivers felt that access to therapies was not provided early enough.[38, 39] They were frustrated with delays in the initiation of rehabilitation after hospital discharge which caused survivors to “go backwards”.[10] Uncertainty about when therapies would start and how arrangements were made, left them feeling abandoned.[50]

The third second order construct related to information. Unmet information needs and gaps in information provision were highlighted in 41% (n = 21) of the studies.[9–11, 25, 27, 28, 35, 37–39, 41, 43–45, 47–51, 55, 62] Opportunities for support could be missed due to the lack of knowledge of what services were available.[28] The lack of information about local services and how to find them was confusing and prevented access.[10, 25, 28, 62] Many caregivers felt that no information was provided at all.[28] Survivors had to find out information by themselves through the internet, friends and other caregivers.[10] When information was provided, it was often inconsistent and covered only some services.[10] Overcoming this gap in information provision required substantial effort: *“I did have to do enormous amount of telephoning*.*”* (caregiver;[25], p. 416). Knowing what help is available, that it can be accessed and telephone contact with a healthcare professional facilitated a caregiving role.[10]

Twelve studies (23%) highlighted insufficient and non-specific information on stroke, its consequences, and recovery.[25, 28, 35, 37–39, 41, 44, 45, 47, 48] Information presented too early after stroke[28] disempowered stroke survivors and caregivers, leading to feelings of confusion, fear[25, 38] and powerlessness.[25] Survivors and caregivers wanted specific information on the significance of post-stroke symptoms (memory loss, swallowing problems, speech, irritability and weight gain) and how to manage them.[48] Lack of information (e.g. on the level of recovery, its trajectory) led to unrealistic expectations of ‘getting back to normal’ given adherence to rehabilitation, leading to disappointment and tensions within a survivor-caregiver dyad.[48] Survivors and caregivers were concerned about medication and wanted to know more about secondary prevention.[11, 27, 49–51]

Eleven studies highlighted aligning information provision to the phase of recovery.[6, 11, 28, 38, 41, 48, 49, 51, 59, 63, 68] Survivors may have limited ability to take in information during the early stages when most information was provided:[49, 51] Caregivers’ information needs increased and diversified with time.[63] More information was needed during preparation for discharge and first few months at home, particularly on long-term rehabilitation goals, secondary prevention, available community services and help navigating the healthcare system.[63] Information on the consequences of stroke (e.g., memory loss, speech difficulties, irritability) was key during post-discharge phase (two months to a year following stroke[48]).

The final second order construct related to quality of communication. Ineffective communication between survivors, caregivers and healthcare services as well as within healthcare services resulted in feelings of frustration[9, 33, 44, 47, 52] and having “to battle the system”.[33] Gaps in the transfer of knowledge within healthcare system[47] and the use of medical jargon sometimes caused confusion[47] and were construed as indifference to survivors’ needs.[52] Insufficient explanations of the therapeutic process during rehabilitation led some survivors to question its efficacy leading to distress and decreased adherence.[47] In contrast, healthcare professionals’ engagement and empathy were valued.[53]

#### Third order constructs

We developed three third order constructs: (1) perceived marginalisation of stroke survivors and caregivers by the healthcare system, (2) passivity and activity in the relationship between patient/caregiver dyad and the services, (3) change and fluidity of needs after stroke of both patients and caregivers.

Perceived marginalisation results from the limited access to healthcare and health interventions after stroke due to the misalignment between how healthcare access in primary care is organised and survivors’ and caregivers’ competencies. Once back in the community, the responsibility to recognise symptoms and to seek care rests with the patient.[69] Accessing healthcare requires mobilisation of individual resources including knowledge of the condition, ability to communicate effectively with healthcare professionals, and awareness of available services.[69] Cognitive and speech and language problems[70–72] can further affect patient’s ability to negotiate healthcare access. The feelings of disappointment and frustration with limited and/or delayed access[10, 37, 38, 54, 58] and the lack of proactive follow-up from healthcare professionals after stroke[6, 11, 12] reflect patients’ (and caregivers’) responses to perceived marginalisation by the primary care and community healthcare services.

**Fig 3:**
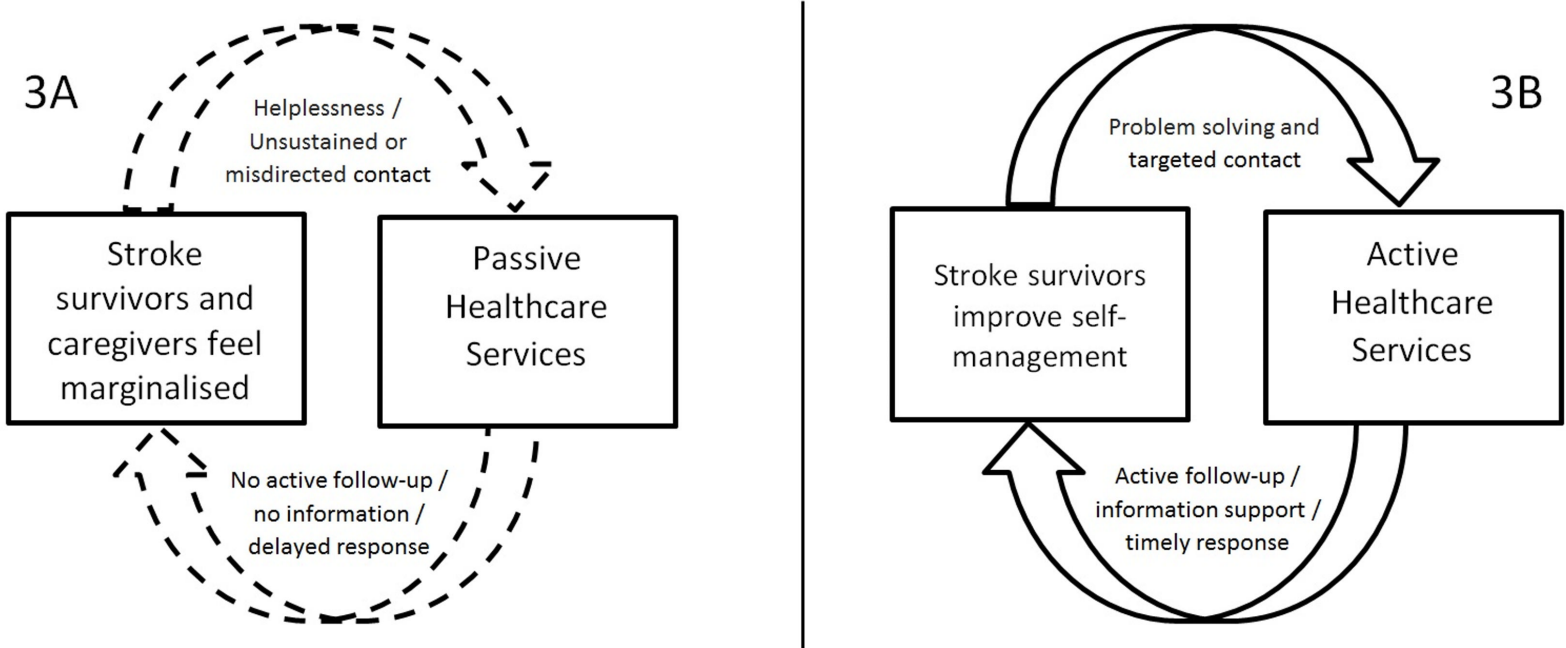
From service passivity and perceived marginalisation to service activity and self-management. 3A: Passive patient-caregiver/ service relationship; 3B: active patient-caregiver / service relationship.

The construct passivity and activity offers a potential solution. It reflects: (1) the tension between passivity of services and the need for proactive service provision to address perceived marginalisation of patients and caregivers by the healthcare system (Fig 3A), and (2) the reciprocity in the healthcare-provider and healthcare-user interactions, where active service provision (e.g. information, follow-up) could encourage active self-management by equipping patients with the necessary tools to better manage the chronic consequences of stroke (Fig 3B). Survivors’[8] and caregivers’[33, 62] active attempts to access information and community services were often met with limited response, including long delays.[62] In one study, the limitations in service provision were construed as barriers set by the healthcare system to ration access.[33] The idea of having to “fight” the (passivity of) the healthcare system[33, 62] can thus be understood as a direct metaphor for perceived marginalisation of stroke survivors and caregivers by the healthcare system. This construct also reflects passivity/activity on the part of the stroke survivor and carer. Perception of marginalisation could stem from lack of support in developing self-management skills. These include: problem solving (e.g. making adjustments in activities of daily living to account for disability), decision making regarding one’s own health (e.g. taking up exercise), finding and utilising resources (e.g. information about condition, recovery and services), forming partnerships with healthcare professionals and taking action (e.g. mastering skills needed to manage the chronic and changing behaviours[73, 74]). Patients and caregivers wanted support with finding and utilising information and relevant healthcare services[62] and looked for guidance from healthcare professionals.[37] Equipping them with these skills could help improve their problem solving and decision making (Fig 3B), especially during the first year when the major adjustment to living with stroke occurs.[75] We posit that the need for active support from services will decrease during the first year after discharge from the hospital as increasing skills and confidence in managing life after stroke will shift the balance towards active patient self-management.

Our final construct, the fluidity and change in (primarily information) needs after stroke emphasises the dynamic aspect of post-stroke recovery. Patients’ needs change with functional improvements (observed earlier in the trajectory) and psychological adaptation to living with stroke.[75] This aspect remained unaddressed by the services. Although information needs diversified and their content changed with time, information provision was not responsive to this change.[6, 11, 51] Caregivers felt that information was not provided at the right time with excess information concentrated in the first few weeks with no follow-up opportunity.[28] The time course of information needs aligns with the need for an ongoing (at least during the first year after stroke) follow-up from a GP.[6, 11, 12]

## Discussion

Stroke survivors and their caregivers feel abandoned because they have become marginalised by services and they do not have the knowledge or skills to re-engage. The marginalisation arises because of service passivity and misalignment of information provision with needs, which change with post-stroke recovery. The passivity of services was expressed as lack of continuity of care, including lack of (active) follow-up, limited (in scope and time) and delayed access to community services, as well as inadequate (too little and too general) information about stroke, recovery and healthcare services. We posit that this passivity also has a relational aspect where activating the support from healthcare professionals within the first year after stroke would increase patients’ ability to self-manage their chronic condition. This can be achieved by providing timely and targeted information about stroke, available resources, and by regular follow-ups to foster supporting long-term relationships with healthcare professionals. Active support from health care professionals would be expected to decrease over time as patients and caregivers become more self-reliant and better able to self-manage living with stroke.

We focussed on post-stroke care delivered by primary and community health services after transfer from specialist services. It is likely that the third order construct of fluidity of need is also relevant to specialist care. For example, the importance of matching information provision to patient and caregiver need.

We identified two key areas for potential service focused interventions to address patients’ and caregivers’ perceptions of marginalisation by the healthcare system after the discharge from the hospital and improve capacity for self-management: (1) increasing stroke specific health literacy by targeted and timely information provision, and (2) improving continuity of care and providing better access to community healthcare services.

### Information provision and increasing health literacy

In alignment with previous work our synthesis identified deficiencies in information provision on several levels: content, format, and timing.[14] Information regarding stroke and recovery was also a common theme in previous reviews.[13, 76, 77] Although qualitative longitudinal studies on the trajectory of stroke recovery are few,[75, 78–80] the insights from such studies could help target both the timing and the content of information provision.[48, 63]

Health literacy encompasses personal skills, ability and motivation of individuals *“(…) to gain access to*, *understand and use information in ways which promote and maintain good health”*([81], p. 357). Our analyses suggest that patients and caregivers want more information about stroke and secondary prevention, and take active efforts to find information for themselves. Previous reviews have highlighted the interface between information provision and utilisation.[13, 82] Large volumes of sometimes conflicting information from multiple providers can impede understanding. What stroke survivors struggled with in our analyses and those by Gallacher et al.[82] is access to sufficient information and the ability to appraise it. Efforts directed towards helping patients identify sources of trustworthy information which are written in an accessible language and format would help increase stroke specific health literacy,[83] which in turn could support better self-management. Third sector initiatives in a number of countries do aim to provide such resources,[84–87] but our analyses indicate that many stroke survivors are not aware of them.

### Continuity of care and support from community services

The lack of continuity of care and its negative consequences for patient care were reported in all previous qualitative reviews which focused on long-term problems,[77] treatment burden,[76] the impact of stroke and its relevance to the development and delivery of services.[13] Three types of continuity can be distinguished: (1) informational (using patient relevant information on stroke and personal circumstances to facilitate appropriate care), (2) management (coherent management of the chronic condition by multiple providers), and (3) relational (an ongoing therapeutic relationship[88]). Unique to our review was a strong emphasis on the lack of follow-up either from a GP, community or specialist services; with patients and caregivers in nine studies (18%) reporting the need for an active follow-up or reassessment to ensure continuity of care.

These two key areas for service interventions could be targeted at the patient, at the carer, or be dyad interventions directed at both patient and carer.[89] We did not review intervention studies in this meta-ethnography. Reviews of evidence of interventions directed at the caregiver and at patient/carer dyads suggest that the former are more effective to improve carer outcome, and the latter to improve patient outcome. [89,90]

### Strengths and limitations

To our knowledge this is the first systematic review on the experiences of stroke survivors and caregivers of primary care and community healthcare services with broad coverage of contexts, samples and study focus. By using meta-ethnography we identified patients’ and caregivers’ perceived marginalisation by the passivity of healthcare services. Importantly, we also identified a relational aspect between service activity and patient self-management thus providing a potential solution for how these perceptions could be addressed. We have included studies from a variety of healthcare contexts (Northern America, UK, Australia, Northern Europe, Iran), involving municipal and rural settings, ethnic minorities,[37, 41, 52, 57, 58] and long-term stroke survivors (62% of the studies included survivors at least 1 year after stroke). The substantial degree of convergence of themes across different settings and samples suggests the transferability of these findings.

Our meta-ethnography focused on experiences of a single patient and caregiver population within a more homogenous healthcare context (primary care and community healthcare services) than in previous reviews. The homogeneity of healthcare contexts we studied facilitated meaningful comparisons and translations across studies in relation to post-discharge and long-term experience of care after stroke.[22] Despite methodological variety (Table 2), all studies included specific themes relevant to survivor and/or caregiver experiences of primary care and community healthcare services.

Potential limitations of qualitative research such as limited generalisability and inability to provide firm answers are offset by the consistency of the findings across epistemological traditions, methodologies, countries and healthcare systems. While the included studies achieved good quality scores, many failed to provide sufficient contextual detail. Only a minority included data on stroke severity, specific long-term impairments (e.g. cognitive impairment, physical disability and aphasia), socio-economic status or ethnicity. Most studies employed a cross-sectional design. Longitudinal studies could provide valuable insights on the temporality and intensity of healthcare needs after stroke relative to the trajectory of recovery. Our synthesis was limited to reports in English. However, only 2% of the studies were excluded based on language and studies from non-English speaking countries (in Northern Europe and Iran) were represented. Our review included studies published up until June 2015. Given that we had over fifty studies, it is unlikely that further third order constructs would be identified by extending the review to the present time–i.e it is likely that we achieved data saturation. We are not aware of major changes in the care offered to stroke patients and their carers in primary care in the last couple of years that might have led to new constructs.

## Conclusions

Primary care and community health care interventions which focus on improving active follow-up and information provision to patients and caregivers especially in the first year after stroke, could help improve patient self-management, increase stroke specific health literacy and thus mitigate the current perceptions of abandonment felt by many stroke survivors and their caregivers.

## Appendix A

### PubMed search strategy

Stroke
Stroke (title/abstract)
1 Or 2
stroke[MeSH Terms]
CVA
cerebral stroke
((stroke) OR Stroke[MeSH Terms]) OR CVA) OR cerebral stroke
patients or survivors or family or caregivers or carers
patients[MeSH Terms]
survivors[MeSH Terms]
family[MeSH Terms]
caregivers[MeSH Terms]
carers[MeSH Terms]
(12) OR 13
(9) OR 10
(11) OR 12
((patients or survivors or family or caregivers or carers) OR 15) OR 16
general practice or family practice
private practitioner or general practitioner or family physician or family doctor
community health services
primary health care
homecare services
primary health care[MeSH Terms]
family physician[MeSH Terms]
general practitioner[MeSH Terms]
private practitioner[MeSH Terms]
family doctor[MeSH Terms]
community health services[MeSH Terms]
general practice[MeSH Terms]
family practice[MeSH Terms]
home care services[MeSH Terms]
(((community health services[MeSH Terms]) OR primary health care[MeSH Terms]) OR family physician[MeSH Terms]) AND home care services[MeSH Terms]
(((general practitioner[MeSH Terms]) OR family doctor[MeSH Terms]) OR general practice[MeSH Terms]) OR family practice[MeSH Terms]
18 OR 19 OR 20 OR 21 OR 22
(32) OR 33) OR 34
perspective or experience or opinion or satisfaction or dissatisfaction or needs or demands
patient satisfaction or attitude or needs assessment
patient satisfaction[MeSH Terms]
attitude[MeSH Terms]
needs assessment[MeSH Terms]
(patient satisfaction[MeSH Terms] OR attitude[MeSH Terms]) OR needs assessment[MeSH Terms]
(37) OR 42
((43) AND 35) AND 17) AND 3
qualitative OR focus group OR interviews
qualitative research
qualitative research[MeSH Terms]
evaluation studies as Topic[MeSH Terms]
focus groups[MeSH Terms]
((((((qualitative) OR focus group) OR interviews)) OR qualitative research[MeSH Terms]) OR evaluation studies as Topic[MeSH Terms]) OR focus groups[MeSH Terms]
(44) AND 51
