# Correction: Stroke survivors' and informal caregivers' experiences of primary care and community healthcare services - A systematic review and meta-ethnography

**DOI:** 10.1371/journal.pone.0196185

**Published:** 2018-04-18

**Authors:** Dominika M. Pindus, Ricky Mullis, Lisa Lim, Ian Wellwood, A. Viona Rundell, Noor Azah Abd Aziz, Jonathan Mant

The number of patients and caregivers included in the study is incorrect in the Results subsection of the Abstract and the third sentence of the Results section. This study included 566 patients and 593 caregivers.
